# Empowered to gain a new foothold in life—A study of the meaning of participating in cardiac rehabilitation to patients afflicted by a minor heart attack

**DOI:** 10.3402/qhw.v10.28717

**Published:** 2015-12-01

**Authors:** Charlotte P. Simonÿ, Pia Dreyer, Birthe D. Pedersen, Regner Birkelund

**Affiliations:** 1 Section of Nursing Science, Institute of Health, Aarhus University, Aarhus, Denmark; 2 Department of quality and education, Slagelse Hospital Region Zealand, Slagelse, Denmark; 3 Department of Anesthesiology, Aarhus University Hospital, Aarhus, Denmark; 4 Research Unit of Nursing, Faculty of Health Sciences, University of Southern Denmark, Odense, Denmark; 5 Section of Health Services Research, Lillebaelt Hospital, Vejle, Denmark; 6 Institute of Regional Health Research, University of Southern Denmark, Odense, Denmark

**Keywords:** Cardiac rehabilitation, coronary heart disease, patients' lived experiences, phenomenological-hermeneutic research, well-being, empowerment

## Abstract

This study aimed to investigate what it means to patients afflicted by a minor heart attack to participate in cardiac rehabilitation (CR). CR is well-established internationally to support patients towards moving forward in satisfying, healthy, and well-functioning lives. Studies indicate that patients achieve improvement in quality of life when participating in CR. However, knowledge of how patients are supported during CR is sparse. Moreover, knowledge of what participating in CR means to patients afflicted by a minor heart attack is lacking. In-depth knowledge in this area is crucial in order to understand these patients’ particular gains and needs. In a phenomenological-hermeneutic frame field observations, focus group interviews, and individual interviews were conducted among 11 patients during and after their participation in CR. Field notes and transcribed interviews underwent three-phased interpretation. It was found that patients were supported to gain renewed balance in their lives during CR. Three themes were identified: (1) receiving a helpful but limited caring hand, (2) being supported to find new values in life, and (3) developing responsibility for the remaining time. The patients were carefully guided through a difficult time and supported to continue in healthy everyday lives. They were given hope which enabled them to find themselves a new foothold in life with respect to their own sense of well-being. This guidance and a sense of hopefulness were provided by heart specialists and more seasoned heart patients. In conclusion, patients were empowered to achieve a healthier lifestyle and improve their personal well-being during CR. However, structural barriers in the programme prevented adequate support regarding the patients’ total needs. Knowledge of the benefits of CR emphasizes the significance of the programme and highlights the importance of high inclusion. Efforts should be made to develop more flexible and longer lasting programmes and further involvement of relatives must be considered.

This paper addresses what it means to patients afflicted by a minor heart attack to participate in a specialized cardiac rehabilitation (CR) programme. This is a part of a larger study aiming to provide knowledge on how patients afflicted by a minor heart attack experience their life situation and the CR programme and what it meant to them to participate in this programme. Knowledge of the patients’ experiences is essential within the area of patient education and care as it raises important issues regarding their particular needs. It is necessary to identify gains and inadequacies in relation to the offered programmes from the patients’ perspective in order to develop sufficient CR with respect to the patients’ health and well-being.

The published literature of the last 20 years has shown that patients with coronary heart disease (CHD) experience sweeping uncertainty featuring changes in mental, social, and physical self-perception (Baldacchino, 2011; Jensen & Petersson, 2003; Johansson, Dahlberg, & Ekebergh, 2003). Research has outlined that patients struggle to regain balance in their lives while managing the consequences of their disease (Fredriksson-Larsson, Alsen, & Brink, 2013; Kristofferzon, Löfmark, & Carlsson, 2007; Tod, 2008). In addition, uncertainty toward the manageable factors has affected patients as they are discharged from the hospital and return to daily life (Bergman & Berterö, 2003; Clark, 2003).

In recent decades, CR has been well-established internationally, aiming to reduce risk factors, improve prognosis, and support the patients in achieving overall psychosocial well-being in their lives (Mampuya, 2012). Differences in the pathology of the presentation of CHD have led to separate diagnostic subcategories and different recommended pathways of treatment (Thygesen et al., 2012). For example, patients afflicted by a minor heart attack, in terms of unstable angina pectoris (UAP) or non-ST-elevation myocardial infarction (NSTEMI) are included in the same fast-track pathways in Denmark recommended by the guidelines set forth by the Danish Health and Medicines Authority (Figure 1). Often these patients are treated invasively and discharged within a few days after being diagnosed. Subsequently, they are enrolled in outpatient CR.

**Figure 1:**
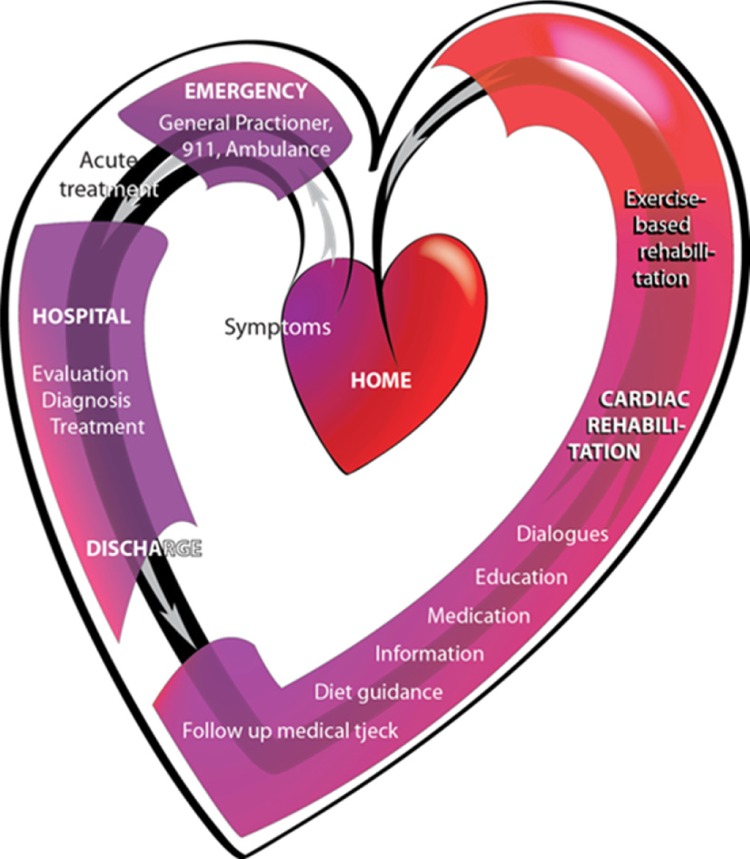
Pathway in the standard treatment for patients with UAP or NSTEMI in Denmark. The heart symbolizes a journey which the patients experience according to the findings of this study. The pathway is structured as a flowchart according to the guidelines from the National Heart Plan (Sundhedsstyrelsen, 2013). When CHD is suspected, acute admission to the hospital is initiated as the administration of medicine and a search for a diagnosis is started. Within 72 h after the onset of symptoms, the coronary arteries are examined by percutaneous coronary interventions (PCI), and angioplasty or stenting are performed if relevant and possible. Discharge from hospital often occurs 1–3 days after the invasive procedure. Subsequently, outpatient cardiac rehabilitation (CR) for approximately 12 weeks is offered at the hospital. In Region Zealand (where this project took place), CR consists of exercise-based cardiac rehabilitation (ECR) offered as a physiotherapist-guided joint physical training (8 weeks); individual consultations with doctors, nurses, physiotherapists and/or dieticians (based on individual needs); and a group-based psychosocial education session (a 1-day session of 6½ h). Produced by the primary author©.

Based on randomized controlled trials, it was shown that patients feel improvement in their quality of life in terms of physical, psychological, and social well-being and functional status of their health when participating in CR (Shepherd & While, 2012). A few qualitative interview studies have also shown that patients regain an increased sense of well-being and confidence to return to enjoyable activities from CR (Jones, Greenfield, & Jolly, 2009; Keaton & Pierce, 2000). However, new studies indicate that it is still a great challenge to move on in life with CHD (Alsén, Brink, Persson, Brändström, & Karlson, 2010; Najafi Ghezeljeh, Yadavar Nikravesh, & Emami, 2014), and knowledge of the meaning of the current and specialized CR to the patients is lacking.

Unfortunately, many patients drop out of CR, and barriers to better adherence are related to the fact that CR does not sufficiently address the patients’ specific situations (Redfern et al., 2012; Sonne, Voss, Kilsmark, Søgaard, & Würgler, 2012). Thus, of paramount importance is the need for firmly addressing CR to be more responsive to the patients’ perceived needs (Dubouloz et al., 2010; Pâquet, Bolduc, Xhignesse, & Vanasse, 2005). We must take into account the experiences of the persons living with specific subcategories of CHD in order to develop attractive CR. In 2015 we published findings of patients’ lived experiences of exercise-based CR (ECR). Here we presented that patients suffering from a minor heart attack experienced an existential anxiety with respect to their sick heart. However, during the ECR the patients overcame this and gained renewed self-efficacy which made them able to continue in an active and satisfying everyday life (Simonÿ, Pedersen, Dreyer, & Birkelund, 2015). The present paper moves a step further and outlines the findings of what participating in the CR means to these patients.

## Methods

To investigate the patients’ experiences, we conducted a qualitative study consisting of ethnographic field observations, semi-structured focus group (FG) interviews, and individual interviews. Field notes (FN) and transcribed interviews were interpreted using a three-phased phenomenological-hermeneutic approach. By using these three methods it was possible by a “bridled attitude” to investigate what it meant to the patients to participate in the CR programme (Dahlberg, 2006). The primary author is a former cardiac nurse and by this “bridling” the phenomena that were meaningful to the patients were investigated with what is seen as the most open mind possible (Dahlberg, 2006; Dahlberg & Dahlberg, 2003). However, this openness is always embedded in the researcher's world and should therefore be seen in the light of the pre-understandings which are briefly illustrated in the section of background.

The approach included the formation of first impressions during the data generation. When it was possible such initial impressions were further examined in order to explore in-depth what it was about.

### Setting and participants

Data generation was performed by the primary author in a Danish hospital from May 2012 to April 2013. Nine men and two women were included (Table I).

**Table I:** Demographic data of the participants.

Participant	P1	P2	P3	P4	P5	P6	P7	P8	P9	P10	P11
Participated in	FN1	FN1	FN2/FG1	FN2/FG1	FN2/FG1	FN2/FG1	FN2/FG1	FN3/FG2	FN3/FG2	FN3/FG2	FN3/FG2
Sex	♂	♂	♀	♂	♂	♂	♀	♂	♂	♂	♂
Married/cohabiting	+	+	+	+	−	+	+	+	+	+	+
Age	64	62	62	74	63	64	66	63	63	87	59
Diagnosis	NSTEMI	NSTEMI	UAP	NSTEMI	NSTEMI	NSTEMI	NSTEMI	NSTEMI	NSTEMI	UAP	UAP
Known CHD, month	1	2	5	1	1	2	2	1	14	1	8
Job status	R	SL	W	R	SL	PSL	R	PSL	SL	R	R

The patients who enrolled in the study were assigned codes, such as P1, P2, P3, up to P11. The table shows the demographic data of the patients and the field notes (FN) and focus group interviews (FG) in which they participated. All of the patients participated in individual interviews (I). Their job status is presented according to the following: retired (R), sick-listed (SL), partly sick-listed (PSL) or working (W). Produced by the primary author©.

Patients were included if they were enrolled in the UAP/NSTEMI-CR, spoke and understood Danish, and did not suffer from other competing life-threatening diseases. The patients attended 8-week-long group-based CR programmes (Table II). The primary investigator included the patients in the study and was given full access to them during their hospitalization and the rehabilitation-programme. She was neither an active part of the CR team nor the care in the ward, but she knew the staff.

**Table II:** The set-up for the group-based cardiac rehabilitation.

Ten to fifteen patients with various ischemic diagnoses were enrolled in group-based CR, typically within 1–4 weeks after discharge from hospital. The programme took place in the hospital and consisted of shared physiotherapist-guided exercise-based training for 1½ h twice a week for 8 weeks and one 6½-h-long session of psychosocial education, which was held around midway in the programme. Most of the patients brought their closest relatives in the psychosocial education session.		

The concept of the exercise-based sessions	The concept of the psychosocial education session	
The training took place in a large training room. It consisted of:	In a meeting room lectures were held as follows:	
Warm-up exercise for approximately 10–15 min followed by spinning, running, varied training with workout equipment, and games, like dodge ball or hockey; we finished by stretching out or deep breathing relaxation exercises. Well-known music	**8:30 am–10:00 am**	**What is ischemic heart disease?** **- About arteriosclerosis, medicine and psychological reactions** Performed by a nurse.
with quick stimulating rhythms was often used in the warm-up and the training. During stretch-out and relaxation, peaceful or pan flute music was played.	**10:15 am–11:00 am**	**Stricken by heart disease** Performed by a hospital priest.
	**11:15 am–12:00 am**	**About the Heart Foundation and life with heart disease** Presented by CHD representatives from the Heart Foundation.
	**Lunch break**	
	**0:30 pm–1:20 pm**	**Exercise** Held by physiotherapists.
	**1:30 pm–2:30 pm**	**The good heart diet** Presented by a dietician.
	**2:40 pm–3:00 pm**	**Social offers** Held by a representative from the local society.

The participants in this study followed three different but similar group-based CR programmes, which took place in the hospital and consisted of exercise-based CR and a psychosocial education session. Produced by the primary author©.

#### Field observations

Inspired by ethnography (Hammersley & Atkinson, 2007; Spradley, 1979, 1980), examination of the group-based CR was performed during 90 h. “Participant observation,” alternating between full participation and full observation, was used, combined with ethnographic interviews to openly explore the patients’ lived experiences during the trajectory of CR. Hereby it was possible to investigate how the patients expressed themselves over time, both in bodily terms and by means of statements, how they interacted with each other and the heart specialists, how they responded to the programme, and what was meaningful to them. During the observations notes were written in a notebook. Immediately after the sessions FN were written with focus on what was expressed by the patients and how it was revealed (Emerson et al., 1995, pp. 26–30; Spradley, 1980, p. 78). Weekly summaries of what was found were made and it was continuously decided whether something should be investigated further by focused mini tour observations (Spradley, 1980). The FN consisted of 265 pages.

#### Focus group interviews

FG interviews were performed on the day that the group-based CR ended. The purpose was to let the patients disclose what was meaningful to them during the trajectory of CR through open discussions. The interviews were moderated so that the patients could share, question, and challenge perspectives in a dynamic and trusting setting (Malterud, 2012). A semi-structured interview guide was developed to reflect the patients’ experiences. As it was aimed to investigate what it meant to the patients to participate in CR, the following main questions were included: “How did you experience the CR offered in the hospital?” and “What has it meant to you to participate in the CR?” When using this guide it was emphasized that the patients should reveal their experiences by their own concepts and common language (Kvale & Brinkmann, 2009; Wilkinson, 1998). The interviews commenced with a session where the participants were asked, one by one, to present themselves and briefly describe what being afflicted by a minor heart attack had meant to them. This was to have everyone be a part of the discussion from the start. The guide was used to ensure coverage of the aim. When other relevant issues came up, they were explored. The interviews were closed when it was ensured that all of relevant issues had been mentioned. Two FGs were conducted, consisting of either five or four patients. Each session lasted approximately 1½ h. The recorded FG interviews were transcribed into a total of 62 pages.

#### Individual interviews

The patients were interviewed individually 1–2 months later. The aim was to gain more insight into what was meaningful to the patients during and after participating in the CR, through private stories (Fog, 2004; Kvale & Brinkmann, 2009). To create a relaxed and attentive atmosphere, the interviews were conducted in a room at the hospital or in the patients’ private homes, according to their wishes. The same semi-structured interview guide as was used in the FG interviews also guided the individual interviews Again room was given to other relevant issues. Follow-up questions were asked to explore preliminary impressions from field observations and FGs at the end of the interviews. For example it was asked: “You earlier expressed that it was rewarding to participate in the CR, can you elaborate on this?” or “You all agreed that it was nice to hear presentations from more seasoned heart patients. Please describe your experience of that?” Each interview lasted approximately 1 h and was ended only when it was ensured that the patients had nothing more to add. The recorded individual interviews were transcribed into a total of 178 pages.

### Ethical considerations

According to Danish law, the study did not need to apply for scientific permission because of its non-biomedical character. It was reported to the Danish Data Supervisory Committee by Region Zealand (“Para syd60” J. no. 1-01-83-0030-08) and their requirements for safe storage of data were followed. The recommendations of the Declaration of Helsinki (World Medical Association Declaration of Helsinki, 2000) were followed and the ethical guidelines for nursing research in the Nordic countries were met (Sygeplejerskers Samarbejde i Norden, 2003). The participants received oral and written information about the study and gave written consent. Regarding the field observations and especially the FG interviews, the patients agreed to be discrete about each other.

### Analysis and interpretation

A phenomenological-hermeneutic approach was used to interpret the data, which consisted of 505 pages of written FN and transcribed interviews. Inspired by the French Philosopher Paul Ricoeur's theory of interpretation (Ricoeur, 1976), we conducted three analytical interpretation phases to reveal the meaning of the patients′ experiences. These phases included naïve understanding, structural analysis, and critical interpretation and discussion. According to Ricoeur, this method benefits from the dialectic movement between explanation and understanding and provides an understanding of what the text as a whole addresses (Dreyer & Pedersen, 2009; Ricoeur, 1976).

#### Naïve understanding

With as open a mind as possible, the whole text was read several times to achieve an initial understanding of what it was about. We let the text speak to us while capturing what it disclosed about what it meant to the patients to participate in the CR. Ricoeur emphasises that this phase is important, but constitutes only a preliminary component of the overall interpretation that must be validated by subsequent structural analysis.

#### Structural analysis

We structured and explained the text by units of meaning (what is observed/what is said) and units of significance (what is the observation about/what is being talked about). To ensure coherence, we compared the units of meaning and the units of significance with the naïve understanding. In this way, we recognized three themes that formed the foundation of the core finding which was that *patients were supported to find a new foothold in life during CR*. An example is presented in Table III. It should be noted that no theoretical perspectives guided this analysis.

**Table III:** An example of the structural analysis.

A part of the structural analysis regarding the finding: Supported to find a new foothold in life during cardiac rehabilitation		

Units of meaning: “What is said/what is observed”	Units of significance: “What is being talked about/what is the observation about”	Theme:
“It has been a truly positive event for me to participate in the training and to have that particular nurse that I spoke with in the hospital. It was really good because then I felt that I could see some light at the end of the tunnel” (I P11).	Patients were well supported to change habits and organize their everyday tasks in better ways.	Supported to find new values in life
“It was a good way to build up some mental sturdiness” (I P5). “I appreciated this rehabilitation. It made me look upon some things with different eyes” (I P 11). They stand in a cluster and talk about the two experienced patients from the heart foundation in the psychosocial education yesterday. The presentation had been very entertaining and with good points. In an admiring voice P3 says that it was really rewarding to listen to people that are suffering from heart disease themselves. Hearing about how they have experienced it all. The other consents by nodding or saying “Hmm.” P5 smiles and says that they had made him all high. They agree that it was good to hear how the more seasoned heart patients had tackled their problems with the disease. They talk about the fact that it was fine to hear a person who talked about the stages he had undergone. In addition, they agree that the best thing was to see that they were doing so well. That their everyday lives had become good again (FO2).	The heart specialists and more seasoned heart patients helped the patients to trust in their future and to find new meaningful ways to thrive in their everyday lives. The patients were guided to focus on their particular needs and to find out what was important and realistic to them. This made them prioritize more consciously. The patients considered the process of finding new values as a strengthening one.	
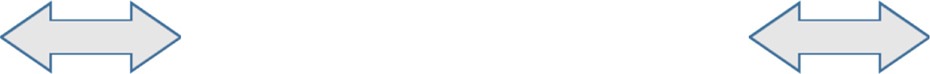		
“It made me get some thinking going. There is no doubt that if I had not participated down here, we would not have started doing exercise as we did. Moreover, we had probably not been able to change our eating habits. In this way, it has been a help.		
In addition, one becomes more aware of some things and is trying to get a little more energy during the day. Changing work a little so that you get some breaks or what to call it. Finding time to do some other things. Say ‘**this** must also be done’ (with emphasis in his voice). Before it was perhaps more like that the job came first rather than some other things … now I am more aware of these things. … My family and I would like to spend our time differently than before and maybe do more of what we desire” (I P6).		
“It went in and really touched you, so that you started thinking a little more about whether you actually took good enough care of yourself” (I P9).		
“It was emphasised that you could build onto the experience with this particular disease and break unto the other side and really feel that things had become better” (FG2 P8).		

Following the method described by Pedersen (2005) the text was structured and explained by units of meaning and units of significance. On the basis of these units themes were identified. The arrows indicate that the process of structuring units of meaning and units of significance and identifying themes can be characterized as dialectic, because this analysis moves forwards and backwards among these three stages to substantiate the basis and argument for the themes (Pedersen, 2005).

#### Critical interpretation and discussion

Given the themes, the findings were further interpreted and discussed with the theory of empowerment, philosophy of hope and personal development, and other research. Here, the interpretation moved from the individual to the general (Pedersen, 2005).

## Findings

### Naïve reading

The overall impression from the naïve reading was that the patients were well-supported through a difficult time towards a renewed positive balance in their lives during CR. After being afflicted by heart disease, the patients were very uncertain. They felt that their freedom of action had been limited and it was a demanding task for them to find proper ways to cope. However, through the CR programme, the patients were guided to find new and meaningful ways to enjoy their everyday lives. They were given useful advice regarding medicine and lifestyle but they were also supported to tackle what challenged them in their personal everyday lives. Even so, the patients felt that the programme should have been longer and better structured. Moreover, they would have liked their relatives to be more involved in the ECR.

### Structural analysis

In the following structural analysis the naïve reading is validated and explained through the three themes found: (1) receiving a helpful but limited caring hand, (2) being supported to find new values in life, and (3) developing responsibility for the remaining time. The themes are based on the disclosures expressed by the patients. The presentation includes direct quotations from FN, FG and individual interviews (I).

#### Receiving a helpful but limited caring hand

It was clear that the patients experienced being carefully guided through a tough time in life during the CR. They felt supported in a helpful way by the heart specialists to overcome a challenging uncertainty. One male patient said: “It is great the way they are offering a helpful caring hand right from the start” (I P8). This underlines how the patients experienced the CR as a valuable help.

The patients clearly expressed feeling secure under the guidance of the physiotherapists. In the field observations it was observed that the patients initially became scared when their pulse beats increased or they became short of breath or felt dizzy when training. The physiotherapists taught them what were normal effects from training and when they needed to take a break and give time to let the pulse slow down and investigate whether they had angina. In most cases the patients realized that they were just having normal effects of healthy training. However, in a few cases some suffered from angina. From these situations they all learned how to handle it by sitting or lying down using nitroglycerin and calling for health professionals if the angina was persistent. One patient stated:

They guided us to train in appropriate ways. They pointed out for example in the spinning training what you could do and when you should take a break. And I learned that I was capable of much more than I had imagined. (I P3)

Another said, while the others were nodding in agreement:

You get this great feeling because they keep an eye on you. They keep a close check on your breathing and whether you can endure the strain when running. They were really in control of it. And they told you to slow down if it was necessary. (FG2 P9)

Another patient said: “I felt that they pushed you to keep on running if you were capable” (FG2 11).

In addition the patients often described to the physiotherapists what they had experienced at home regarding, for example, feeling high pulse beats during the night. The physiotherapist guided them to perform exercises of relaxation and explained to them how to calm down and find rest in their bodies by deep breathing and letting the body feel the gravity. The patients learned to achieve bodily and mental peace by these excises, and often they asked particularly for this to be performed at the end of the training session. From the ECR it is observed that they enjoyed performing relaxation on mattresses, as in the following example:

They follow the guidance of the physiotherapist and the calming pan flute music fills the room. They seem very relaxed and breathe deeply with closed eyes. When finished, they rise slowly with red cheeks and calm smiles on their faces. They chat while smiling and laughing as they leave the training room. (FN2)

These examples cover how the patients felt guided safely to interpret the signals from their own bodies and how to both exercise appropriately and find peace in their bodies.

The patients expressed that they appreciated the consultations with the nurse because she guided them in handling their uncertainness regarding life with a sick heart. They explained that they were given advice regarding the administration of their regular medicine and how to prevent undue straining of the heart by taking nitroglycerin before they were going to exert themselves physically. Some patients were also given advice in relation to the planning of travels abroad, which they feared especially due to worries of having another heart attack when being abroad. “It was a great relief to speak to the nurse. And you could talk to her about everything. You could just ask and she knew all the answers” (I P11). This quote shows how the patients appreciated this as a useful support when trying to understand and tackle all the different aspects of their new situation. The patients described that they felt well taken care of by the heart specialists, who treated them with respect. This was clearly expressed this way: “She listens and smiles and talks to me about how I feel. She helps me with my concrete matters. It is really nice seeing her, and I am always looking forward to the next consultation” (FO3 P9). Another patient said:

She was really straightforward and brought up issues about sexual activity. She said that I should just take a whiff of this thing, [produces a nitroglycerine spray] and then I would be ready again. (I P11)

These statements illustrate how the patients felt respected as the human beings they thought themselves to be and were supported to feel more personal confidence during their struggle to continue in their everyday lives with the sick heart. Moreover the patients praised the fact that they could call their nurse and discuss concreate matters when they needed. In sum, the guidance from the heart specialists worked as a helpful caring hand to the patients with respect to their personal needs.

However, it turned out that the patients felt that this helpful care was too limited. It was clear that it was a challenge for the patients to follow the entire CR programme. Some were not able to attend all sessions because they were too tired or feeling unwell, as one patient explained: “I have not been well enough to be able to train” (I P2). The patients emphasized that their consultations were often scheduled to take place in the middle of a training session. They said that they regretted missing the valuable help from the heart specialists when they had to skip sessions. In addition, some expressed that they would need a longer programme: “I would say that something is missing when the last consultation with the nurse has ended” (FG2 P9) “When you no longer have direct access to specialists, then you feel all alone in the world” (FG2 P11). These quotes illustrate how the patients were concerned when the guidance by the heart specialists came to an end. It made them feel left to themselves with the responsibility of handling living with the disease. They felt uncertain whether they were capable of handling this responsibility.

#### Being supported to find new values in life

During the CR, the patients often talked with the heart specialists about how they could change habits and organize their everyday tasks in better ways. It was a difficult effort for the patients, but they expressed that they felt well-supported in this process. For example one patient said: “It was a good way to build up some mental sturdiness” (I P5). Another patient stated it this way:

It has been a truly positive event for me to participate in the training and to have that particular nurse that I spoke with in the hospital. It was really good because then I felt that I could see some light at the end of the tunnel. (I P11)

These examples cover how the patients felt supported by the heart specialists to find new, meaningful ways to thrive in their everyday lives and to trust in their future.

The patients had great benefits from the psychosocial education session during which they were supported in focusing on their particular needs. One male patient described it as follows: *“*It went in and really touched you, so that you started thinking a little more about whether you actually took good enough care of yourself” (I P9). The example confirms how the patients experienced that they were guided towards a sharper focus on themselves. To the patients, it meant that they *got some thinking going* and began to change their attitude towards what was important to them. Both during field observations and interviews the patients expressed that they had become more aware of how they could obtain more well-being in their everyday lives if they did not overestimate their energy and choose to do things which they enjoyed. It made them prioritize more consciously so that they readjusted their ambitions to a more realistic level concerning tasks such as work and housekeeping. One patient explained how he changed focus after being guided in the CR: “My family and I would like to spend our time differently than before and maybe do more of what we desire” (I P6).

Clearly it was of great importance to the patients to hear contributions from more seasoned heart patients, who during the psychosocial education session told them about their personal way of handling the challenges of their disease. It was obviously rewarding to hear how others had overcome the uncertainties and changes that they had been through. The patients paid particular attention to the indications they received from the more seasoned heart patients that they had gotten along well with the passing of time. It made them more optimistic about their own situation. This is clearly expressed in a field observation where the patients evaluated the presentation: “They agreed that the best thing was to see that they were doing so well. That their everyday lives had become good again” (FO2).

In addition the patients expressed that the psychosocial education had left them an overall positive impression of their possibilities in life. One man explained it this way: “It was emphasized that you could build onto the experience with this particular disease and break unto the other side and really feel that things had become better” (FG2 P8). The quote confirms how the patients realized that the process of finding new values after having been afflicted with CHD could be a strengthening one and that through the education in the CR they were given an important hope for the future.

#### Developing responsibility for the remaining time

The study showed that the patients often talked to the heart specialists as well as to their peer patients about how their attitude to life had changed. During the CR programme, the patients became particularly good at reminding each other of the fact that they should appreciate every new day that was given to them. They agreed on being thankful to have survived the heart attack. It made them look at life itself in a new light, which one female patient described as follows: “I have begun to see every day as a gift” (I P3). Also along these lines, the patients said that during the trajectory of CR they developed a responsibility to do something meaningful with the time that was left to them. For example, they explained that they learned to prioritize events that made themselves and their relatives happy. The patients also described that they were given inspiration on realizing some of their dreams, even if an element of risk was included in doing so. As one example a male patient for a long period had been sitting at home fearing doing anything. After having heard the more seasoned heart patients in the education session he stated: “What has happened shall not make me refrain from travelling. I still have places that I would like to visit. I must see China before the bloody thing breaks down again [points to his heart]” (I P5). This illustrates how the patients were encouraged to make important decisions during the CR.

The patients explained how during the CR they learned that they had *built up for the disease over many years* and that they felt that they had been given a warning that something ought to be changed. During both the ECR and the education session it was highlighted to the patients that physical inactivity, stress, and eating unhealthy food were predictors for cardiovascular disease. In a discussion in the education session one male stated it this way:

I have been eating too much and too fat. Unfortunately I have not done much exercise. And now when I have been given this broad hint, I must say that if I do not do anything about it, then I would think of myself as being bloody ungrateful. (FN1 P1)

This quotation covers how the patients came to understand the mechanisms of the heart disease and became motivated to change their way of living so that they took better care of their health during ECR. In line with this, the patients made an effort to follow the instructions given by the heart specialists, urging them to start exercising and eating wholesome food. It was clear from their experience that a more active lifestyle increased a pleasant feeling of being healthy, which was expressed in the following statement:

All of a sudden it is all about your life. I have realised that it is of importance for your quality of life. And if you read a bit about it, you come to see that those who are active have a better chance of becoming older than those who do not exercise. (I P 11)

The patients explained how being physically active, which they learned from the ECR, made them feel that their energy, self-esteem, and quality of life improved. From this perspective, the patients became focused on the importance of being in a healthy physical condition during CR.

However, the patients also described that their relatives were worried about their heart being able to stand the physical strain and often asked them to hold back a little with hard physical work or training. The patients explained that they would have liked to have been able to bring along their relatives to the ECR so that they could have observed with their own eyes how much strain people with CHD can actually put into their activities. The patients stated that this was very important because they needed their relatives to stop being overprotective and instead support them in making an effort towards improving their health and well-being by being physically active and continuing effective training.

## Discussion and conclusions

### Critical interpretation and discussion

The findings show that patients afflicted by a minor heart attack during CR received guidance that helped them understand the disease and how to cope with it in proper ways. This supported them to overcome a demanding initial uncertainty and made them capable of leading a more satisfactory everyday life with physical activity and better awareness on their personal needs. This is in line with other recent studies that showed that cardiac patients achieve increased knowledge and behaviour changes through educational interventions (Ghisi, Abdallah, Grace, Thomas, & Oh, 2014; Sol, Van der Graaf, Van Petersen, & Visseren, 2011) and that CR and ECR improve self-care and ability to reduce risk factors by incorporating an active lifestyle (Chatziefstratiou, Giakoumidakis, & Brokalaki, 2013; Heran et al., 2011). It is also supported by a study showing that CR by informational, psychological, and social support was imperative in enabling patients to recover from acute coronary syndrome (Pryor, Page, Patsamanis, & Jolly, 2014). However, to our knowledge, the present study is the first to describe how the heart specialists provide careful guidance to the patients so they can learn what works for them as individuals.

The most novel finding was that the patients were not only supported to cope with the medical situation and follow recommendations of lifestyle but also to find new values in their lives by focusing on their own needs and sense of well-being. The present findings illuminate how the patients received care that addressed their personal needs in ways that enhanced their ability to make decisions in their lives. Moreover, the patients developed a responsibility for their remaining span of time to be both healthier and more worthwhile with respect to their personal well-being. Thus the CR seemed to provide the patients with empowerment. The concept of patient empowerment draws from the field of education and is often related to Paulo Freire's theory about education for critical consciousness (Freire, 1996, 2005). Within this line of thought, the aim of education is to increase the learner's freedom and autonomy so that he or she can be in power of their own lives. By trustful and careful dialogue, the learner should be supported to gain the capacity to adapt to reality and to make choices that transform this reality (Freire, 1996). Freire describes this as a movement from naïve consciousness to critical consciousness that leads to critical action (Freire, 2005).

The findings of the careful guidance provided by the heart specialist and the development of the patients’ ability to act on behalf of their own health priorities and values can thus be interpreted as showing the CR as an educational programme leading to critical consciousness and action. In this light, the patients truly became in power of their own lives via the CR. They were supported to make choices that draw from information yet at the same time are in accordance with the patients’ own sense of well-being. This is in line with other studies examining empowerment in relation to diabetes care (Anderson & Funnell, 2005, 2010). To the best of our knowledge, the empowerment approach is a new dimension in the CR. However, it seems to be very useful as the findings indicate that the patients developed a greater sense of self-efficacy during the CR. They were supported to move on in life being more physically active and with better focus on their personal needs.

This study also shows that the patients are given hope for their future life. The heart specialists and the more seasoned patients help them to see that they can overcome their struggles and have a satisfactory life with the heart disease. Furthermore they also teach the patients that the process they are going through can be a strengthening one. According to the Danish philosopher Søren Kierkegaard, a person's reality can change when serious sickness occurs, which may lead to despair. Such despair includes, at least for some time, the lack of being able to act in accordance with one's true self (Kierkegaard & Thielst, 2011). In the present study the uncertainness that the patients struggle with can be seen as such despair. Kierkegaard emphasizes that hope is the only antidote to despair. Hope is to expect the good in one's life. In his thinking, one person's hope can be edifying to another so that the trust in a good future can be seen as a possibility (Kierkegaard, 2013). In this light, the hope that the patients were given from the heart specialists and the more seasoned heart patients is very essential because it supported them in expecting the good in life and conquering the despair that the heart disease had caused. This encouraged them to be open to the possibilities in their new situation and find out how they could move on, despite the changes it included. In this way they were provided with help to find their new true selves. Using Kierkegaard's terms they are supported to gain a new understanding of themselves and thus find a new foothold in life, with the disease (Jf. Kierkegaard & Thielst, 2011). This indicates that the patients are supported in an existential way during the CR.

The findings further showed that the patients had difficulties attending the entire CR programme, which they regretted. Poor adherence to CR is a problem because it might decrease outcome regarding lifestyle changes and possibilities to achieve and maintain optimal psychosocial and physical health. Other studies have outlined that the lack of interest and misconceptions regarding CR are key barriers for attending CR (McKee et al., 2014; Redfern et al., 2012). In contrast, this study emphasizes that the patients find it challenging to attend the whole programme due to the programme set-up. In addition we found that the patients needed the CR to be longer, which, according to another study of Danish CR, could include considerable potential regarding mental health and cardioprotective drugs (Larsen, Vestergaard, Søndergaard, & Christensen, 2011). Considering the challenging processes that the patients go through, according to the present study, longer lasting CR with extended support could be beneficial.

Last, we found that the patients asked for more information for their relatives concerning the ECR. They needed their relatives to understand that they were capable of doing exercises so that they did not prevent the patients from doing so. Other studies show that relatives of myocardial infarction (MI) patients share a feeling of existential threat and that couples’ daily living is affected mainly due to dread of another heart attack (Jensen & Petersson, 2003; Svedlund & Danielson, 2004). With this in mind, it should be considered whether the relatives need more information and involvement in the ECR.

## Conclusions

In CR, patients afflicted by a minor heart attack are empowered to lead a life with a healthier lifestyle and more well-being. With respect to their sick hearts they become able to move on in a physically active everyday life. In addition they learn to have a better focus on their personal well-being. During the CR programme the patients find new values for themselves and develop a responsibility for their remaining span of life to be healthy and worthwhile. They are supported by both heart specialists and more seasoned heart patients to follow this in their daily living. The patients are carefully guided and provided with hope through a difficult time where they struggle learning to live life with heart disease. The impact is that the patients are supported to find themselves a new foothold in life when participating in CR. However, CR did not meet all the needs of the patients. There were structural barriers for full attendance; a longer programme with extended help and care could improve the support to the patients and further involvement of relatives in the ECR was needed.

### Study limitations and strengths

The present findings are specific to patients afflicted by a minor heart attack and the Danish specialized CR for these patients. However, in the right context, comparisons to what it means to cardiac patients to participate in CR can be made regarding patients with other CHD diagnoses or participation in different CR programmes.

A combination of participant observations and FG as well as individual interviews provided very detailed and rigorous insight into the phenomena that were expressed by the patients during the whole CR programme and during the initial time afterwards. It allowed us to achieve insight by an open and “bridled attitude,” which is valuable when the patients’ perspective is sought. We found that validation across the whole data material was possible. What was observed could be recognized in statements from interviews and vice versa. And most importantly, the observations and interviews supplemented each other so that more adequate insights were gained.

Within the phenomenological-hermeneutic approach it is a premise that there is not only one way to interpret a text (Ricoeur, 1976). Therefore, there has been a sustained critique of the steps and components of the analysis, by the author group, confirming that the interpretation most likely reflected the reality that the patients expressed. In addition, the findings were discussed with other colleagues in the field to obtain conformability. Representative quotes from the data were presented to provide credibility and to illustrate the findings.

### Practice implications

Knowing that empowerment to leading a satisfactory life with improved well-being can be gained through CR accentuates the importance of including as many as possible in the programme. Efforts must be made to inform patients of these gains to increase attendance. This might also facilitate the implementation of similar programmes where they have not yet been established. Though the offer was valued as positive by patients, it was clear that there is still room for improvement. Thus, whether more flexible and perhaps longer offers should be developed must be considered. It must be taken into account whether the relatives should have more information and if there are other ways in which the patients’ individual needs can be met.
